# Psychological flourishing among young and middle-aged postoperative lung cancer patients: a latent profile analysis

**DOI:** 10.3389/fonc.2026.1973408

**Published:** 2026-10-16

**Authors:** Xiuxiu Wang, Yaxuan Gui, Yingying Sang, Tuo Zheng, Huihui Fan, Dan Su, Baoming Wang

**Affiliations:** 1 Department of Thoracic Surgery, Anhui Chest Hospital, Hefei, China; 2 School of Nursing, Anhui Medical University, Hefei, China; 3 Nursing Department, Anhui Chest Hospital, Hefei, China; 4 The Taikang Health and Wellness Industry Research Institute, Anhui Medical University, Hefei, China

**Keywords:** flourishing, hope, illness perception, latent profile analysis, lung cancer, social support, young and middle-aged adults

## Abstract

**Background:**

Young and middle-aged patients after lung cancer surgery often bear multiple psychological pressures caused by illness and daily life. As a vital positive psychological resource, flourishing may help improve their postoperative psychological adaptation. This study aimed to identify the latent profiles of flourishing among young and middle-aged patients after lung cancer surgery, examine the characteristics of each profile and explore factors associated with these profiles.

**Methods:**

Between August 2025 and June 2026, a cross-sectional survey was conducted on 404 young and middle-aged postoperative lung cancer patients from a tertiary specialized hospital in Anhui Province. Data were collected using general demographic questionnaire, Flourishing Scale (FS), Herth Hope Index (HHI), Social Support Rating Scale (SSRS), and Brief Illness Perception Questionnaire (BIPQ). Latent profile analysis was employed to identify distinct flourishing profiles, and multinomial logistic regression analysis was used to explore factors associated with profile membership.

**Results:**

The four-class model demonstrated optimal fit (entropy=0.806, LMR *p* = 0.022, BLRT *p* < 0.001). Four distinct latent flourishing profiles were identified: Moderate optimistic flourishing subtype (22.03%), Moderate interpersonal flourishing subtype (27.97%), Low flourishing subtype (28.22%), and High flourishing subtype (21.78%). Compared with the low flourishing profile, higher emotional response scores were associated with the moderate interpersonal profile (*OR* = 1.273, 95%*CI* = 1.094~1.480). Higher social support and emotional response scores were associated with the high flourishing profile (*OR* = 1.126, 95%*CI* = 1.017~1.248; *OR* = 1.221, 95%*CI* = 1.038~1.437). No variables were associated with the moderate optimistic profile.

**Conclusion:**

This study identified four latent profiles of flourishing among young and middle-aged postoperative lung cancer patients, demonstrating substantial heterogeneity in positive psychological adaptation toward postoperative disease stress. Illness-related emotional responses and social support show distinct associations with profile membership. Medical institutions should deliver stratified and refined psychological management according to each patient’s specific characteristics. Future longitudinal studies may explore the dynamic trajectories of these profiles.

## 1 Introduction

Lung cancer ranks first in both incidence and mortality worldwide and in China, imposing a substantial global and domestic disease burden (1, 2). Driven by the popularization of early screening programs, lung cancer is increasingly diagnosed at younger ages, with individuals aged 18–59 years constituting a pivotal patient subgroup (3). Radical surgical resection serves as the primary curative intervention for early-stage lung cancer, effectively eliminating lesions and prolonging survival (4). Nevertheless, postoperative trauma, persistent rehabilitation stress, and persistent fear of disease recurrence collectively create cumulative psychological strain, restricting patients’ social role adaptation and impairing their overall quality of life (5). Compared with elderly survivors, young and middle-aged lung cancer patients carry heavier familial and occupational responsibilities. A cancer diagnosis may drastically disrupt their social identity and elicit persistent psychological distress, which substantially undermines their postoperative psychological adjustment (6, 7). Therefore, it is vital to explore the psychological status of this population.

The Implementation Plan for Cancer Prevention and Control under the Healthy China Initiative (2023-2030) underscores the necessity of integrated full-cycle cancer management and highlights unmet psychosocial care demands among cancer survivors (8). Beyond negative emotional responses triggered by malignancy, patients may also develop adaptive positive psychological outcomes, defined as psychological flourishing within positive psychology frameworks (9). Flourishing refers to a holistic, optimal state of mental well-being (10). For postoperative cancer survivors, flourishing transcends general subjective well-being. It represents an adaptive psychological resource. It involves constructing personal meaning, sustaining interpersonal bonds, maintaining rehabilitation confidence, and achieving self-acceptance. These processes occur amid surgical trauma, prolonged treatment burden, and disrupted social roles (11). Patients with high flourishing levels exhibit enhanced resilience against postoperative physical discomfort, career interruption, and household obligations, alongside reduced symptoms of anxiety and depression and superior long-term quality of life (12). Existing research has preliminarily explored psychological flourishing among maintenance hemodialysis patients (13). Most current psychological research on lung cancer populations focuses on negative emotions such as anxiety and depression while few studies explore positive psychological indicators represented by flourishing (14, 15).

The social support buffering model holds that external social support can alleviate illness stress to a certain extent and facilitate patients to develop adaptive coping strategies (16). Hope serves as a vital internal positive resource. It mitigates illness uncertainty and helps patients establish positive beliefs for rehabilitation (17). Illness perception refers to patients’ subjective cognition of disease progression and prognosis, which exerts impacts on individuals’ psychological adjustment (18). Social support, hope and illness perception participate in the psychological adaptation process of cancer patients from the dimensions of external resources, internal beliefs and cognitive evaluation. Prior studies have verified the close correlations between the three variables and the mental status of cancer survivors (18, 19). Nevertheless, the effects of these three factors on psychological flourishing among young and middle-aged lung cancer patients remain unclear and deserve further exploration.

Most traditional psychological research adopts variable-centered approaches, which reflect population-average relationships but cannot capture heterogeneity among individuals. Latent profile analysis (LPA) is a person-centered statistical method. It sorts participants into mutually exclusive subgroups based on response patterns of observed indicators. It has been widely applied to identify psychological subtypes among oncology patients (20, 21). At present, few studies have adopted LPA to explore psychological flourishing profiles for postoperative lung-cancer patients. Therefore, this study aimed to identify latent flourishing profiles of young and middle-aged postoperative lung cancer patients and explore related factors so as to provide reference for stratified psychological intervention in clinical practice.

## 2 Methods

### 2.1 Participants

This cross-sectional study recruited participants from a tertiary hospital in Anhui Province, China through the convenience sampling method. The study was reported in accordance with the Strengthening the Reporting of Observational Studies in Epidemiology (STROBE) guidelines (22). (**Supplementary Material S1**) Between August 2025 and June 2026, a total of 404 patients with lung cancer among young and middle-aged populations were invited to participate in the study. The inclusion criteria were as follows: (1) aged 18–59 years old, following the age criterion used in previous studies on young and middle-aged cancer patients in China (23); (2) diagnosed with primary lung cancer by pathological examination and underwent radical lung cancer surgery; (3) stable postoperative condition, clear consciousness with normal reading, comprehension and communication capabilities; (4) informed consent obtained and volunteered to take part in this investigation. The exclusion criteria were as follows: (1) individuals grappling with severe heart, liver, kidney dysfunction or other malignant tumors; (2) individuals with cognitive impairment, mental disorders, or communication difficulties.

### 2.2 Sample size

Based on the recommendations of Nylund-Gibson and Choi (24), a minimum sample size of 300 participants is recommended for latent profile analysis to ensure adequate identification of smaller latent profiles. Additionally, general guidelines for multivariate analysis suggest that the sample size should be at least five times the number of observed variables (25). In this study, the number of observed variables includes 11 demographic variables, 8 flourishing items, 11 hope level items, 10 social support items and 9 illness perception items, resulting in a total of 49 observed variables. Thus, the formula for sample size is 245, which means that at least 245 subjects are needed for this study. Also, the sample size should be further expanded considering the sample loss rate of 20%. Therefore, the minimum sample size required is 307. The final sample comprised 404 participants, which exceeded the required threshold and was therefore deemed sufficient for the planned analyses.

### 2.3 Measurements

#### 2.3.1 General information questionnaire

The general information questionnaire was self-designed by the researchers on the basis of literature review, including sex, age (years), marital status, educational level, occupational status, per capita monthly household income (CNY), medical payment mode, chronic comorbidities, surgical approach, pathological type, tumor stage, lymph node dissection.

#### 2.3.2 Flourishing Scale

Flourishing was measured using the FS originally developed by Diener et al. (26). The scale was translated and culturally adapted into Chinese by Lai (27). It comprises eight items measuring self-perceived success in domains such as meaning, self-acceptance, relationships, helping others, engagement, competence, optimism, and being respected by others. Respondents rate each item on a 7-point Likert scale ranging from 1 (strongly disagree) to 7 (strongly agree), with total scores ranging from 8 to 56, where higher scores indicate greater psychological flourishing. In this study, the Cronbach’s α coefficient for the scale was 0.804.

#### 2.3.3 Herth Hope Index

Hope levels were measured using the Chinese version of the HHI, originally developed by Herth K (28) and translated and culturally adapted into Chinese by Zhao et al. (29). The scale consists of 12 items and 3 dimensions: temporality and future, positive readiness and expectancy, and interconnectedness. Items are rated on a 4-point Likert scale ranging from 1 (strongly disagree) to 4 (agree). The total score ranges from 12 to 48, and higher scores indicate greater hope. The Chinese version of the HHI has demonstrated good reliability in cancer patients, with a reported Cronbach’s α of 0.971 (30). The Cronbach’s α coefficient of the scale in this study was 0.836, and the Cronbach’s α coefficients of each subdimension across groups were 0.727, 0.728 and 0.735, respectively.

#### 2.3.4 Social Support Rating Scale

This study used the SSRS developed by Xiao to measure participants’ social support level (31). The scale consists of 10 items and 3 dimensions: subjective support, objective support, and utilization of support. Items 1–5 and 8–10 are rated on a 4-point Likert scale ranging from 1 (not at all) to 4 (very much). Items 6 and 7 are multiple-response items, each listing eight potential sources of social support. If “No source” is answered, a score of 0 is assigned; if any source is selected, each chosen source is assigned 1 point. The maximum score for each of these two items is 8 points. The total score ranges from 12 to 66, and higher scores indicate higher of social support level. The Cronbach’s α coefficient of the scale in this study was 0.836, and the Cronbach’s α coefficients of each subdimension were 0.715, 0.706 and 0.757, respectively.

#### 2.3.5 Brief Illness Perception Questionnaire

This scale was originally developed by Broadbent et al. (32). and subsequently translated and culturally adapted by Mei et al. (33). It is designed to evaluate patients’ subjective perceptions of illness threats. The scale consists of 9 items covering four dimensions: emotional responses (2 items), illness identity (1 item), understanding (1 item), and cognitive representations (5 items). Items 1 to 8 are scored on a 0–10 scale, with total scores ranging from 0 to 80. The ninth item (illness identity) is an open-ended question and is not included in the total score. This item can collect qualitative text data but cannot generate continuous scores. Previous studies have also excluded this item from quantitative analysis (34). Higher scores indicate stronger subjective perceptions of illness threats. The Cronbach’s α coefficient of the scale in this study was 0.782. The Cronbach’s α coefficients were 0.851 and 0.736 for emotional responses and cognitive representations, respectively.

### 2.4 Data collection

On-site questionnaire surveys were conducted by uniformly trained investigators. Standardized instructions were provided to inform patients of the research purpose, questionnaire filling requirements and confidentiality principles. Questionnaires were completed anonymously by participants independently. All questionnaires were collected on the spot and checked for completeness; participants were invited to supplement missing items in a timely manner. A total of 436 questionnaires were distributed. Thirty-two questionnaires were excluded due to invalid responses, including 9 with incomplete information and 23 where respondents selected the same option for more than 80% of all items. Finally, 404 valid questionnaires were obtained, with an effective response rate of 92.66%.

### 2.5 Data analysis

The data were analyzed using SPSS 26.0 software and Mplus 8.3 software. First, latent profile analysis was conducted with Mplus 8.3 software to explore patterns of flourishing levels among young and middle-aged postoperative lung cancer patients. Scores from the 8 items of the Flourishing Scale were served as manifest variables, and models with 1 to 5 categories were sequentially fitted. Robust maximum likelihood estimation was adopted for model estimation. Class-invariant variances were specified. Random starts were set to 1000 200. This specification helped reduce the risk of local maxima. Model convergence was carefully examined. All models terminated normally. The best loglikelihood value was replicated across random starts. No negative residual variances were observed. The optimal number of profiles was determined based on a comprehensive evaluation of model fit indices and the theoretical interpretability of each solution. Lower values for the Akaike Information Criterion (AIC), Bayesian Information Criterion (BIC), and adjusted BIC (aBIC) indicated better model fit (23). Entropy values closer to 1 signified higher classification accuracy. *p* < 0.05 in the Lo-Mendell-Rubin (LMR) likelihood ratio test or the Bootstrap-based likelihood ratio test (BLRT) indicated that the current category model outperforms the previous-level category model. Second, the data were statistically analyzed using SPSS 26.0. Normality was assessed via the Kolmogorov-Smirnov test, histograms and Q-Q plots, which confirmed that measurement data did not conform to a normal distribution. Measurement data were expressed as medians with interquartile ranges, while count data were expressed in terms of frequency and percentage. For univariate analysis, the Kruskal-Wallis *H* test was employed to examine the measurement data, the chi-squared test or Fisher’s exact test was used for unordered categorical data, and the Kruskal-Wallis *H* test was utilized for ordered categorical data. Multinomial logistic regression was performed to identify factors associated with latent profile membership, incorporating variables with statistical significance in univariate analyses. Differences were considered statistically significant at *p* < 0.05.

### 2.6 Ethics consideration

This study was conducted in accordance with the ethical principles outlined in the Declaration of Helsinki. This study was approved by the Medical Ethics Committee of Anhui Chest Hospital (Approval No. KY-2026-112-01). Trained researchers explained the study objectives, survey procedures, and research significance to each eligible patient during face-to-face interviews. All participants provided written informed consent after being fully informed that participation was voluntary and anonymous. They were assured of their right to withdraw at any time without restriction, and that such withdrawal would not negatively affect their routine clinical care or follow-up. All participants were assured that their data would remain confidential, accessible only to the research team, and used solely for academic purposes.

## 3 Result

### 3.1 Sociodemographic characteristics of participants

A total of 404 valid questionnaires were included in the final analysis, and the participant enrolment flow is presented in **Figure 1**. Most participants were middle-aged, with patients aged 45–59 years accounting for 73.02% of the total sample, and females making up 60.15%. The vast majority of respondents were married (95.30%), diagnosed with non-small cell lung carcinoma (96.78%) and stage I-II early lung cancer (98.02%). In terms of educational attainment, 63.61% of participants had a senior high school education or below, 56.44% were employed, and 98.76% of patients were covered by employee or resident medical insurance.

**Figure 1:**
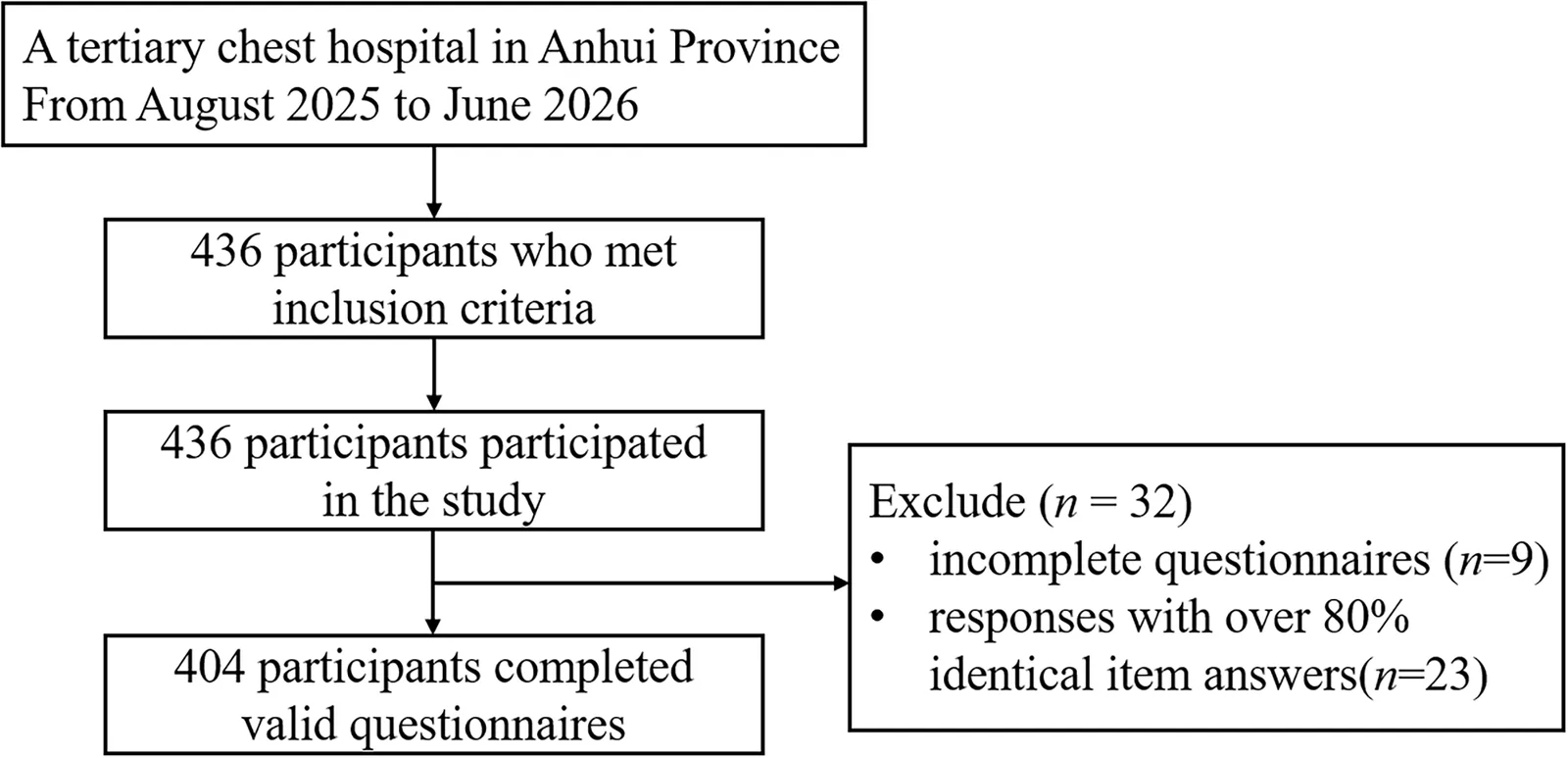
Flowchart of participant enrolment.

### 3.2 Selection of optimal latent profile model

The best-fitting LPA was the four-profile model. The fit indices for models with one to five latent profiles are presented in **Table 1**. The AIC, BIC, and aBIC values decreased progressively up to the four-profile model. The five-profile model showed a further decrease in these fit indices (**Figure 2**; **Supplementary Table 1**). However, the LMR likelihood ratio test for the five-profile model showed no statistical significance (*p* = 0.158). Moreover, the five-profile model contained a minor subgroup of only 14 participants, representing 3.47% of the overall sample. This small subgroup size is associated with limited clinical representativeness. The four-profile model exhibited an entropy value of 0.806 (>0.80). This value falls within an acceptable range. The four-profile model also showed superior model parsimony and clearer clinical interpretability. In addition, the average posterior classification probabilities across the four latent subgroups were 87.9%, 86.2%, 92.6% and 92.0%, respectively, which further validated the stability. Notably, Profile 2 was relatively lower, but above 80% (**Table 2**).

**Table 1:** Model fit indices for latent profile analysis of flourishing among young and middle-aged postoperative lung cancer patients.

Class	AIC	BIC	ABIC	Entropy	LMRT(P)	BLRT(*P*)	Category probability(%)
1	7724.643	7788.666	7737.896	–	–	–	–
2	7169.818	7269.853	7190.525	0.784	<0.001	<0.001	59.41/40.59
3	7022.909	7158.957	7051.071	0.775	<0.001	<0.001	27.18/49.88/22.94
**4**	**6922.579**	**7094.640**	**6958.196**	**0.806**	**0.022**	**<0.001**	**22.03/27.97/28.22/21.78**
5	6873.114	7081.187	6916.185	0.837	0.158	<0.001	3.47/33.17/ 22.28/20.79/ 20.30

AIC: Akaike information criterion; BIC: Bayesian information criterion; aBIC: adjusted Bayesian information criterion; LMRT: Lo-Mendell-Rubin adjusted likelihood ratio test; BLRT: Bootstrapped likelihood ratio test.

Bold values indicate the best-fitting model.

**Figure 2:**
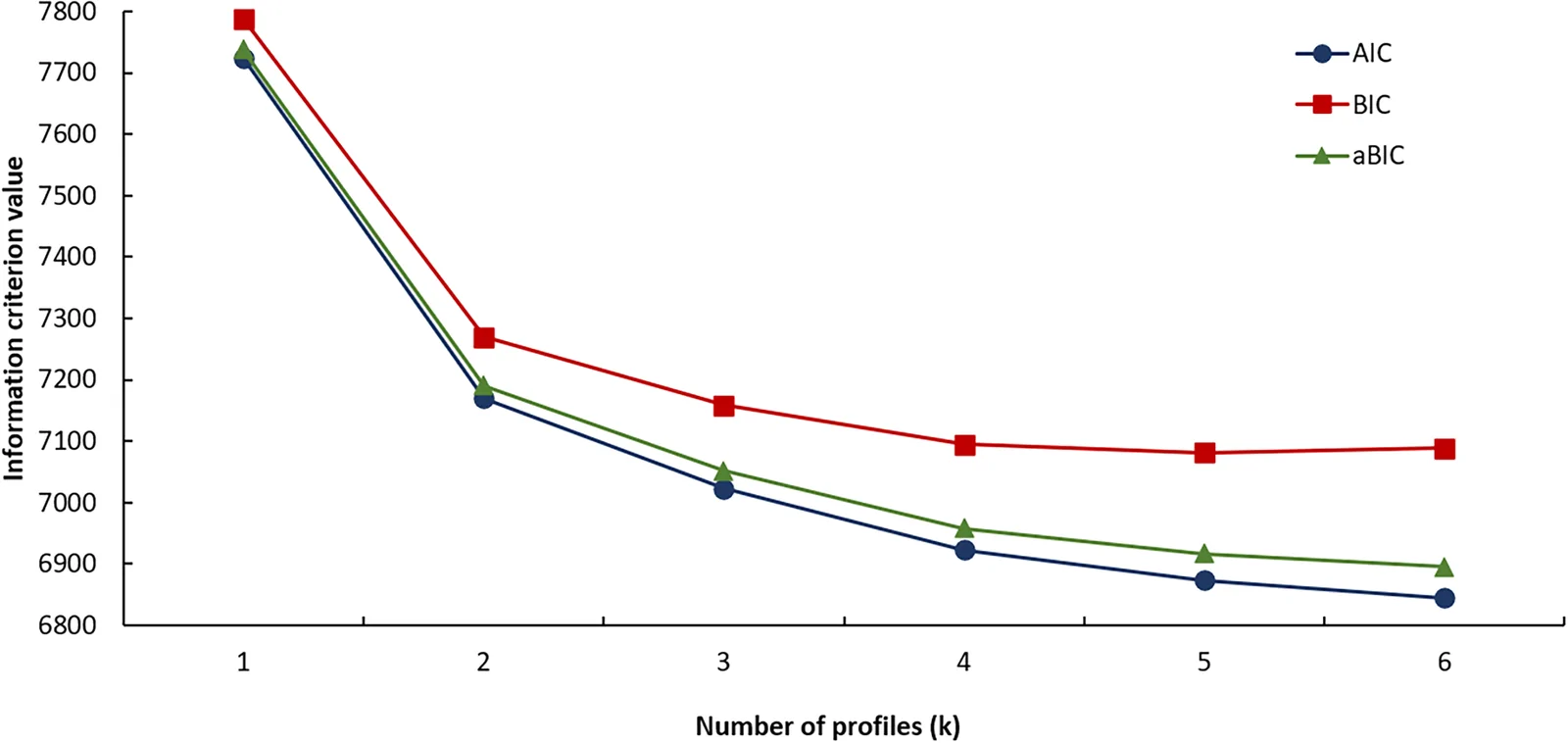
Scree plot of model fit indices for latent profile solutions.

**Table 2:** Average attribution probability of each latent class (Column) for samples assigned to each latent profile (Row).

Category	C1	C2	C3	C4
C1	0.879	0.065	0.034	0.022
C2	0.041	0.862	0.076	0.021
C3	0.011	0.063	0.926	0.000
C4	0.039	0.041	0.000	0.920

### 3.3 Characteristics and designations of each latent profile

According to the eight dimensions of the FS, latent profile discrimination was performed in this study. **Figure 3** illustrates the distributional tendencies of average scores across each dimension of the FS. Among the four-profile solution identified, Profile 1 comprised 22.03% of the sample and exhibited moderate flourishing. Optimism emerged as the most salient dimension. This profile was designated the “Moderate optimistic flourishing” subtype. Profile 2 comprised 27.97% of the sample and exhibited moderate flourishing. The interpersonal dimension emerged as the most salient dimension. This profile was designated the “Moderate interpersonal flourishing” subtype. Profile 3, constituting roughly 28.22% of the sample and set as the reference group, displays generally lower scores across most FS dimensions, with most indicators sitting at relatively modest levels. This profile is termed the “Low flourishing” subtype. Finally, Profile 4, comprising 21.78% of all respondents, shows comparatively elevated scores across nearly all dimensions of the FS, with most domains reaching relatively favorable levels. This profile is defined as the “High flourishing” subtype.

**Figure 3:**
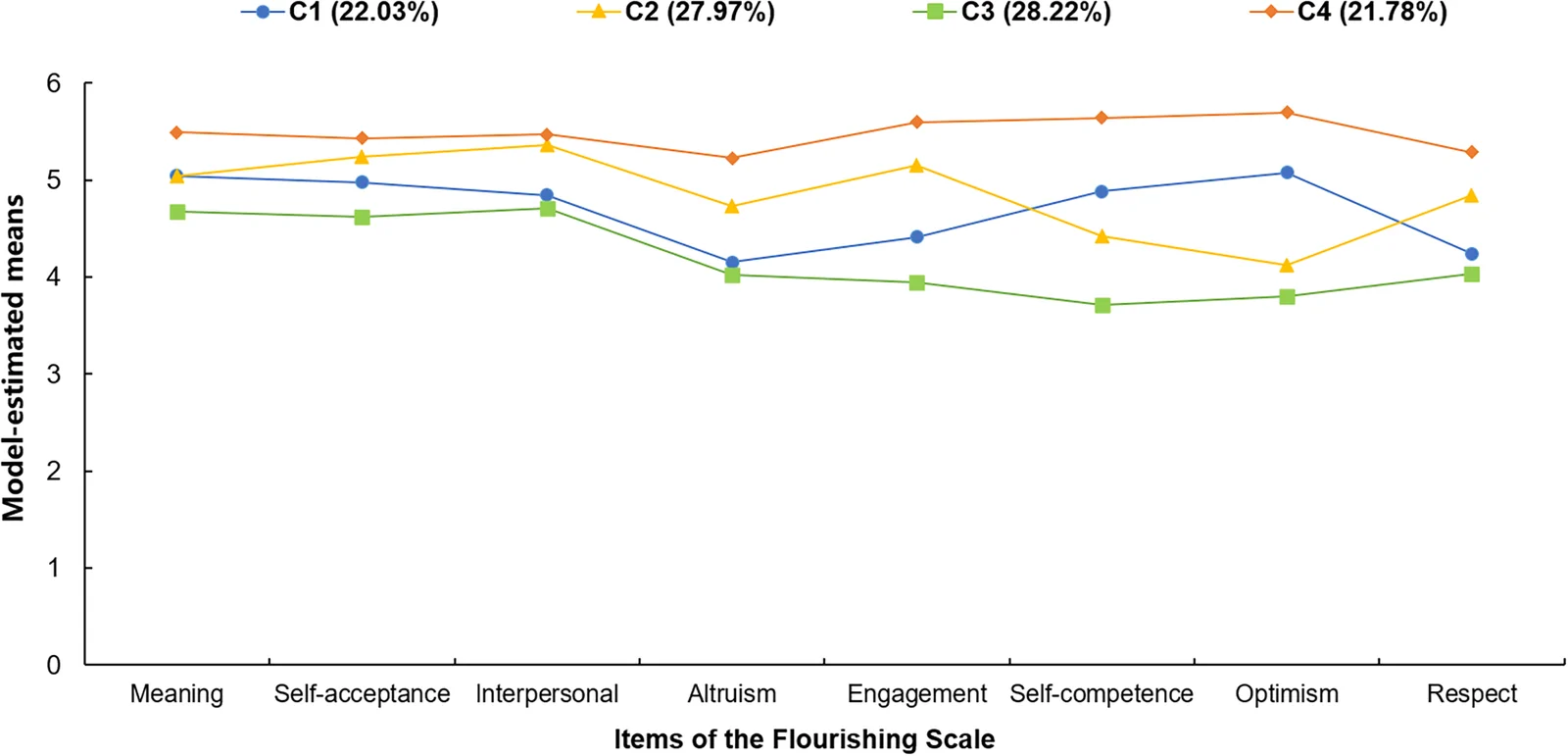
Latent profiles of flourishing among young and middle-aged postoperative lung cancer patients.

### 3.4 Factors associated with membership in flourishing latent profiles

The results of the univariate analysis showed that patients in different flourishing subtypes exhibited significant differences in occupational status, total Hope scores, total social support scores, and the emotional response dimension of illness perception (*p* < 0.05) (**Table 3**) To further investigate these associations, variables that emerged as statistically significant in the univariate analysis were entered as independent variables in a multinomial logistic regression analysis. Prior to model estimation, we conducted a multicollinearity diagnosis. The tolerance values ranged from 0.287 to 0.990, all greater than 0.1. The variance inflation factors (VIF) ranged from 1.010 to 3.480, all of which were below the threshold of 5, indicating that there was no severe multicollinearity (35). The potential profiles of flourishing among participants were set as the nominal dependent variable, with the “Low flourishing” subtype (C3) serving as the reference group. Employment status was coded 1 = employed and 0 = unemployed. Subscale scores of HHI, SSRS and emotional response were incorporated as continuous raw scores.

**Table 3:** Univariate analysis variables associated with latent flourishing profile membership (*n* = 404).

Variables	Latent flourishing profile (*n* (%))				*χ*²*/H*	*p*	Effect size
	C1 (n = 89)	C2 (n = 113)	C3 (n = 114)	C4 (n = 88)			
**Sex**					0.366^a^	0.947	Cramér’s *V=*0.030
Male	33(37.1)	45(39.8)	47(41.2)	35(39.8)			
Female	56(62.9)	68(60.2)	67(58.8)	53(60.2)			
**Age (years)**					2.225^a^	0.527	Cramér’s *V=*0.074
18-44	23(25.8)	32(28.3)	25(21.9)	27(30.7)			
45-59	66(74.2)	81(71.7)	89(78.1)	61(69.3)			
**Marital status**					4.250^b^	0.218	
Married	87(97.8)	107(94.7)	111 (97.4)	81(92.0)			
Unmarried/Divorced	2(2.2)	6(5.3)	3(2.6)	7(8.0)			
**Educational level**					3.077 ^a^	0.380	Cramér’s *V=*0.087
High school or below	55(61.8)	65(57.5)	78(68.4)	53(60.2)			
College diploma or above	34(38.2)	48(42.5)	36(31.6)	35(39.8)			
**Occupational status**					9.890 ^a^	**0.020**	Cramér’s *V=*0.156
Employed	43(48.3)	68(60.2)	57(50.0)	60(68.2)			
Unemployed	46(51.7)	45(39.8)	57(50.0)	28(31.8)			
**Per capita monthly household income (CNY)**					1.091^c^	0.779	*η²_KW_* = 0.000
<3000	14(15.7)	11(9.7)	13(11.4)	12(13.6)			
3000∼5000	42(47.2)	56(49.6)	58(50.9)	44(50.0)			
>5000	33(37.1)	46(40.7)	43(37.7)	32(36.4)			
**Medical payment mode**					2.655 ^b^	0.399	Cramér’s *V=*0.078
Employee/Resident medical insurance	87(97.8)	111(98.2)	114(100.0)	87(98.9)			
Self-pay or others	2(2.2)	2(1.8)	0(0.0)	1(1.1)			
**Chronic comorbidities**					3.718 ^a^	0.294	Cramér’s *V=*0.096
Yes	38(42.7)	52(46.0)	62(54.4)	38(43.2)			
No	51(57.3)	61(54.0)	52(45.6)	50(56.8)			
**Surgical approach**					0.066 ^a^	0.996	Cramér’s *V=*0.013
Wedge resection	42(47.2)	53(46.9)	55(48.2)	41(46.6)			
Pulmonary segmentectomy/Segmentectomy	47(52.8)	60(53.1)	59(51.8)	47(53.4)			
**Pathological type**					1.614^b^	0.711	Cramér’s *V=*0.066
Non-small cell lung carcinoma (NSCLC)	87(97.8)	107(94.7)	111(97.4)	85(96.6)			
Small cell lung carcinoma (SCLC)	2(2.2)	6(5.3)	3(2.6)	3(3.4)			
**Tumor stage**					4.215^b^	0.174	Cramér’s *V=*0.105
Early stage (I/II)	87(97.8)	113(100.0)	110(96.5)	87(98.9)			
Advanced stage (III/IV)	2(2.2)	0(0.0)	4(3.5)	1(1.1)			
**Lymph node dissection**					0.746 ^a^	0.862	Cramér’s *V=*0.043
Yes	30(33.7)	41(36.3)	45(39.5)	33(37.5)			
No	59(66.3)	72(63.7)	69(60.5)	55(62.5)			
**Hope**	40(37,42)	41(38,43)	41(39,42)	43(40,42)	29.748^c^	**0.000**	*η²_KW_* = 0.067
**Social support**	39(35,43)	40(36,44)	40(36,43)	45(40,48)	38.278^c^	**0.000**	*η²_KW_* = 0.088
**Illness perception**	61(57,64)	61(57,65)	59(56,63)	61(57,67)	5.373 ^c^	0.146	*η²_KW_* = 0.006
Cognitive representations	38(35,41)	39(35,41)	38(35,40)	39(37,42)	4.267 ^c^	0.234	*η²_KW_* = 0.003
Understanding	7(6,8)	7(6,8)	7(6,8)	7(6,8)	0.444 ^c^	0.931	*η²_KW_* = 0.000
Emotional responses	16(14,16)	16(14,17)	15(14,16)	16(14,17)	8.742 ^c^	**0.033**	*η²_KW_* = 0.014

a, b and c represent the statistics of chi-squared test, Fisher’s exact test, and Kruskal-Wallis *H* test, respectively. For Kruskal-Wallis *H* tests,*η²_KW_* (eta-squared) was reported as the effect size; For chi-squared tests, Cramér’s *V* was calculated as the effect size. C1: moderate optimistic flourishing profile; C2: moderate interpersonal flourishing profile; C3: low flourishing profile; C4: high flourishing profile.

Bold values indicate statistically significant associations (p < 0.05).

Compared with the “Low flourishing” subtype, higher emotional response scores were positively associated with the “Moderate interpersonal flourishing” subtype (*OR* = 1.273, 95%*CI* = 1.094~1.480). Additionally, higher social support and emotional response scores were positively associated with the “High flourishing” subtype (*OR* = 1.126, 95%*CI* = 1.017~1.248; *OR* = 1.221, 95%*CI* = 1.038~1.437). All included variables showed no statistically significant associations with the “Moderate optimistic flourishing” subtype. The regression results are presented in **Table 4**.

**Table 4:** Multinomial logistic regression of potential profiles of flourishing (*n* = 404).

Variables	C2: Moderate interpersonal flourishing profile			C4: High flourishing profile		
	*β*	*p*	*OR* (95%*CI*)	*β*	*p*	*OR* (95%*CI*)
Intercept	-6.108	0.006	–	-9.008	0.000	–
Unemployed (Ref: Employed)	-0.336	0.225	0.715[0.415, 1.230]	-0.527	0.092	0.590[0.320, 1.090]
Hope	0.058	0.384	1.060[0.930, 1.208]	0.024	0.761	1.024[0.879, 1.192]
Social support	0.004	0.919	1.004[0.924, 1.092]	0.119	**0.023**	1.126[1.017, 1.248]
Emotional responses	0.241	**0.002**	1.273[1.094, 1.480]	0.200	**0.016**	1.221[1.038, 1.437]

The low flourishing profile (C3) was defined as the reference category for multinomial logistic regression. All included variables showed no statistically significant associations with classification into the moderate optimistic flourishing profile (C1) (all *p* > 0.05) The multinomial logistic regression model: *χ²* = 56.824, *df* = 12, *p* < 0.001. Nagelkerke pseudo-*R²* = 0.140. McFadden pseudo-*R*² = 0.051. Overall classification accuracy = 39.4%.

Bold values indicate statistically significant associations (p < 0.05).

## 4 Discussion

### 4.1 Heterogeneity of flourishing among young and middle-aged postoperative lung cancer patients

Four latent profiles of flourishing were identified in this study. These profiles reflect the heterogeneity of positive psychological adaptation among young and middle-aged postoperative lung cancer patients. This finding was consistent with the previous study by Huang et al, who observed heterogeneous latent patterns of spiritual well-being in patients with early-stage lung cancer (20). Such heterogeneity suggests that a single intervention protocol may not fit all patients, and the intervention target may depend on the profile of each patient.

The low flourishing profile was the largest subgroup (28.22%) and had the lowest scores on almost all dimensions of the FS. This result suggests that some young and middle-aged postoperative patients are in a generally suboptimal state of positive psychological functioning. Previous studies have shown that psychosocial distress in cancer care changes across the cancer care continuum, and that distress screening needs to be repeated during cancer care (36). In lung cancer populations, symptom burden and anticipatory grief are associated with lower positive psychological functioning and meaning in life (37). Therefore, interventions for patients in the low flourishing profile may address multiple dimensions of positive functioning at the same time and may include strengthening psychological resilience (38). Symptom burden is associated with demoralization through family function, resilience, and coping behaviors (39). For this subgroup, early screening for distress and symptom burden, together with resilience-oriented support, may have clinical value.

The moderate optimistic flourishing profile (22.03%) was characterized by relatively higher optimism scores but relatively lower interpersonal connection scores. This result suggests that these patients have moderate levels of positive psychological resources, while their relational resources are relatively insufficient. Previous studies have shown that spiritual well-being is associated with illness perception in patients with lung cancer (40). For this profile, interventions may preserve existing optimistic strengths while addressing interpersonal communication and connection.

The moderate interpersonal flourishing profile (27.97%) showed the opposite combination, with relatively stronger interpersonal connection but relatively lower optimism, self-acceptance, and meaning. This result suggests that interpersonal resources alone may not be sufficient for comprehensive flourishing. Evidence from young and middle-aged patients with lung cancer after surgery shows that social support, health literacy, and perceived control are associated with health-promoting behaviors (41). Therefore, interventions for this profile may preserve interpersonal strengths while addressing optimistic beliefs, self-acceptance, and meaning construction.

The high flourishing profile (21.78%) showed relatively balanced high scores across all eight dimensions of the FS, together with higher levels of internal positive beliefs and external social resources. This result is consistent with previous studies on positive reconstruction of the illness experience (42), and with evidence that positive psychological well-being is a meaningful outcome in cancer survivors (43). These patients may also have better psychological resilience (44). Therefore, interventions for this group may focus on maintaining existing psychological strengths and on monitoring psychological status under postoperative stress.

### 4.2 Factors associated with latent flourishing profile membership

Compared with the low flourishing subtype, higher emotional response scores were positively associated with the moderate interpersonal subtype and the high flourishing profile. This direction contrasts with the conventional assumption that greater illness-related negative emotion corresponds to lower flourishing. Similar paradoxical patterns have been reported in previous research (45). The Common-Sense Model of self-regulation offers a theoretical explanation (46). In this model, emotional representations and cognitive representations operate as partially independent systems. Higher emotional responses may reflect active cognitive processing rather than passive distress. When protective resources are present, this emotional arousal corresponds to adaptive outcomes (47). This finding aligns with the post-traumatic growth framework, in which confronting negative emotions may be a prerequisite for positive psychological reconstruction (45, 48). Thus, the association between higher emotional responses and higher flourishing profiles may represent an adaptive coping pattern. These findings suggest that emotional responses may play a role in positive psychological adaptation. Clinical practice may consider the assessment of illness-related emotional responses in postoperative patients.

In addition, compared with the low flourishing subtype, higher social support scores were positively associated with the high flourishing profile. This suggests that patients with higher levels of social support were more likely to be classified into the high flourishing profile. The social support buffering model holds that external resources can reduce psychological strain under stressful conditions (16). Postoperative lung cancer patients need to cope with rehabilitation stress and social role adjustment, and social support may play a role in this process (48). Specifically, social support may be linked to positive psychological adaptation through pathways such as practical care, access to information, and sharing of illness experiences (49). Previous studies also suggest an association between social support and psychological resilience in patients with lung cancer after surgery (18). Therefore, healthcare providers should evaluate patients’ social support levels during treatment, encourage family emotional support and peer communication, and facilitate the maintenance of positive psychological adaptation.

Hope was not significantly associated with any flourishing profile in the multinomial logistic regression model, although it was statistically significant in the univariate analysis. The attenuation of this association after adjustment suggests that hope may share variance with social support and emotional responses, rather than being independently associated with profile membership. However, previous intervention studies have shown that meaning-oriented spiritual care is associated with improved spiritual well-being in patients with cancer (50). Meta-analytic evidence also supports the effectiveness of positive psychological interventions for well-being in oncology populations (51). Family-oriented dignity therapy is associated with improved spiritual well-being in patients with lung cancer receiving chemotherapy (52). Positive psychological interventions are also associated with improved well-being in patients with a colostomy (53). These findings suggest that although hope was not independently associated with flourishing profiles in this cross-sectional model, meaning construction and spiritual well-being remain clinically meaningful intervention targets.

Notably, no variables showed significant associations with the moderate optimistic subtype, which comprised 22.03% of the sample. This null finding is substantively important. It suggests that the included variables do not fully capture the characteristics of this subgroup. The variables included in the present study (hope, social support, illness perception, and employment status) may not be sufficient to distinguish this group. Other unmeasured variables, such as personality traits, coping style, cancer-related stigma, time since surgery, and psychological flexibility, may be associated with this profile. Psychological flexibility has been shown to be associated with emotional well-being, fatigue, insomnia, and post-traumatic growth in patients with cancer undergoing treatment (54). Therefore, clinical intervention recommendations for the moderate optimistic subtype should be interpreted with caution, and future studies may include additional variables to clarify the characteristics of this subtype.

This study did not record time since surgery or adjuvant treatment status. These two variables may be relevant to psychological flourishing. Patients surveyed shortly after surgery may differ in psychological status from those surveyed several months later, and psychosocial distress may change across the cancer care continuum (36). Patients receiving adjuvant chemotherapy, immunotherapy, or targeted therapy may also report additional symptom burden (39). Because the study period spanned ten months, participants may have differed in recovery stage. Psychological resilience may also change over time in patients with lung cancer (44). The absence of these variables may limit the interpretation of the present findings and should be addressed in future studies.

The sample in this study was also highly homogeneous in several clinical characteristics. Most participants were married, insured, and diagnosed with early-stage NSCLC. This homogeneity may partly explain why marital status, insurance type, pathological type, and tumour stage showed almost no discriminatory power in the univariate analyses and did not differentiate profiles. Because these variables had little variance, they were unlikely to distinguish subgroups of patients with different levels of flourishing, whether or not such differences exist in the broader population. This also means that the profiles identified here should be interpreted in the context of this specific patient group, rather than as a general representation of all young and middle-aged postoperative lung cancer patients. Future studies with more diverse samples are needed to examine whether the same profiles emerge in other patient groups.

## 5 Limitations

This study has several limitations that should be acknowledged. First, all participants were recruited from a single tertiary hospital. The demographic and clinical characteristics of patients from a single medical center cannot fully reflect the overall situation of young and middle-aged lung cancer patients in different regional and medical settings. The sample was also highly homogeneous in this study. Most participants were married, insured, and diagnosed with early-stage NSCLC. These features limit the representativeness and generalizability of the sample. Future multi-center and large-sample studies are warranted to verify and extend the current findings.

Second, this study employed a cross-sectional research design, which does not allow for causal inferences. Longitudinal research designs, such as latent profile transition analysis or latent growth modeling, should be conducted in the future to explore the dynamic developmental trajectories and causal mechanisms of patients’ flourishing.

Third, all psychosocial outcomes relied on self-reported scales, which may introduce social-desirability bias. Furthermore, this study did not measure several clinical variables (eg. symptom burden, cancer-related stigma, time since surgery and adjuvant therapy status). Important variables may have been omitted from the flourishing subtype models. Further research should incorporate more clinical covariates for comprehensive adjustment.

Finally, only surgically treated lung cancer patients were enrolled, whereas patients receiving non-surgical palliative therapy were excluded. Therefore, the research findings cannot be generalized to inoperable patient populations. Additionally, the age cutoff of 59 years follows Chinese national convention. This cutoff may differ from international definitions of young and middle-aged adults, which may limit the cross-cultural generalizability of the findings.

## 6 Conclusion

Young and middle-aged postoperative lung cancer patients present four heterogeneous latent profiles of flourishing. Illness-related emotional responses and social support show distinct associations with specific flourishing subtypes. Clinical practice may target emotional regulation and social support enhancement. Medical institutions may consider delivering stratified intervention strategies according to each patient’s specific characteristics. These strategies may support precision psychological care after surgery. Future longitudinal research may further explore the dynamic development of these profiles and contribute to full-cycle management for this population.

## Data Availability

The original contributions presented in the study are included in the article/**Supplementary Material**. Further inquiries can be directed to the corresponding authors.
