# Supplementary material for: Psychological flourishing among young and middle-aged postoperative lung cancer patients: a latent profile analysis
Source: Front Oncol. 2026 Oct 16;16:1973408. doi: 10.3389/fonc.2026.1973408 (PMC13719391; doi:10.3389/fonc.2026.1973408)
Supplement: Supplementary file 1 [file Table1.docx]

**Supplementary material S1**

**STROBE Statement**

|  | **Item** | **Recommendation** | **Page** | |
| --- | --- | --- | --- | --- |
| **Title and abstract** | 1 | (*a*) Indicate the study’s design with a commonly used term in the title or the abstract | 1-2 | |
|  |  | (*b*) Provide in the abstract an informative and balanced summary of what was done and what was found | 2-3 | |
| **Introduction** | | | | |
| Background/  rationale | 2 | Explain the scientific background and rationale for the investigation being reported | 3-4 | |
| Objectives | 3 | State specific objectives, including any prespecified hypotheses | 4 | |
| **Methods** | | | | |
| Study design | 4 | Present key elements of study design early in the paper | 4-5 | |
| Setting | 5 | Describe the setting, locations, and relevant dates, including periods of recruitment, exposure, follow-up, and data collection | 4-5 | |
| Participants | 6 | Give the eligibility criteria, and the sources and methods of selection of participants | 5 | |
| Variables | 7 | Clearly define all outcomes, exposures, predictors, potential confounders, and effect modifiers. Give diagnostic criteria, if applicable | 5-7 | |
| Data sources/ measurement | 8* | For each variable of interest, give sources of data and details of methods of assessment (measurement). Describe comparability of assessment methods if there is more than one group | 5-7 | |
| Bias | 9 | Describe any efforts to address potential sources of bias | 7 | |
| Study size | 10 | Explain how the study size was arrived at | 5 | |
| Quantitative variables | 11 | Explain how quantitative variables were handled in the analyses. If applicable, describe which groupings were chosen and why | 7-8 | |
| Statistical methods | 12 | (*a*) Describe all statistical methods, including those used to control for confounding | 7-8 | |
|  |  | (*b*) Describe any methods used to examine subgroups and interactions | 8 | |
|  |  | (*c*) Explain how missing data were addressed | 7 | |
|  |  | (*d*) If applicable, describe analytical methods taking account of sampling strategy | / | |
|  |  | (*e*) Describe any sensitivity analyses | 9 | |
| **Results** | | | | |
| Participants | 13* | (a) Report numbers of individuals at each stage of study—eg numbers potentially eligible, examined for eligibility, confirmed eligible, included in the study, completing follow-up, and analysed | 9 | |
|  |  | (b) Give reasons for non-participation at each stage | 9 | |
|  |  | (c) Consider use of a flow diagram | 9 | |
| Descriptive data | 14* | (a) Give characteristics of study participants (eg demographic, clinical, social) and information on exposures and potential confounders | 9 | |
|  |  | (b) Indicate number of participants with missing data for each variable of interest | 7 | |
| Outcome data | 15* | Report numbers of outcome events or summary measures | 12-14 | |
| Main results | 16 | (*a*) Give unadjusted estimates and, if applicable, confounder-adjusted estimates and their precision (eg, 95% confidence interval). Make clear which confounders were adjusted for and why they were included | 12-14 | |
|  |  | (*b*) Report category boundaries when continuous variables were categorized | 11-14 | |
|  |  | (*c*) If relevant, consider translating estimates of relative risk into absolute risk for a meaningful time period | 11-12 | |
| Other analyses | 17 | Report other analyses done—eg analyses of subgroups and interactions, and sensitivity analyses | / | |
| **Discussion** | | | | |
| Key results | 18 | Summarise key results with reference to study objectives | 14-18 | |
| Limitations | 19 | Discuss limitations of the study, taking into account sources of potential bias or imprecision. Discuss both direction and magnitude of any potential bias | 18-19 | |
| Interpretation | 20 | Give a cautious overall interpretation of results considering objectives, limitations, multiplicity of analyses, results from similar studies, and other relevant evidence | 14-18 | |
| Generalisabi-lity | 21 | Discuss the generalisability (external validity) of the study results | 17-19 | |
| **Other information** | | | | |
| Funding | 22 | Give the source of funding and the role of the funders for the present study and, if applicable, for the original study on which the present article is based | 20 |  |

**Supplementary Table 1** Comparisons of ΔAIC, ΔBIC, and ΔaBIC across adjacent latent profile models

| Model comparison | ΔAIC | ΔBIC | ΔaBIC |
| --- | --- | --- | --- |
| C1→C2 | 554.825 | 518.813 | 547.371 |
| C2→C3 | 146.909 | 110.896 | 139.454 |
| C3→C4 | 100.330 | 64.317 | 92.875 |
| C4→C5 | 49.465 | 13.453 | 42.011 |
| C5→C6 | 28.957 | -7.056 | 21.502 |

**Supplementary Table 2** Means and standard deviations of flourishing scale items across latent profiles

| Item | C1(M±SD) | C2(M±SD) | C3(M±SD) | C4(M±SD) |
| --- | --- | --- | --- | --- |
| Meaning | 5.02±0.67 | 5.06±0.72 | 4.64±0.53 | 5.50±0.57 |
| Self-acceptance | 4.96±0.69 | 5.27±0.57 | 4.60±0.56 | 5.42±0.58 |
| Interpersonal | 4.82±0.51 | 5.40±0.61 | 4.68±0.52 | 5.47±0.57 |
| Altruism | 4.12±0.65 | 4.71±0.82 | 4.04±0.68 | 5.23±0.72 |
| Engagement | 4.40±0.60 | 5.13±0.71 | 3.94±0.64 | 5.59±0.54 |
| Self-competence | 4.85±0.61 | 4.43±0.68 | 3.68±0.50 | 5.63±0.57 |
| Optimism | 5.10±0.34 | 4.07±0.37 | 3.77±0.46 | 5.69±0.46 |
| Respect | 4.21±0.63 | 4.84±0.73 | 4.02±0.68 | 5.31±0.70 |

**Supplementary Table 3** Overall Kruskal-Wallis tests and post-hoc pairwise comparisons of flourishing scale items across latent profiles

| Item | Overall test  (*χ*²) | C3 vs C1(*z*) | C3 vs C2(*z*) | C3 vs C4(*z*) | C1 vs C2(*z*) | C1 vs C4(*z*) | C2 vs C4(*z*) |
| --- | --- | --- | --- | --- | --- | --- | --- |
| Meaning | 78.617  *p<*0.001 | 3.931  *p=*0.001 | 4.764  *p<*0.001 | -8.834  *p<*0.001 | -0.539  *p=*1. 000 | -4.640  *p<*0.001 | -4.369  *p<*0.001 |
| Self-acceptance | 90.835  *p=*0.001 | 3.663  *p*<0.001 | 7.462  *p*<0.001 | -8.558  *p=*0. 005 | -3.333  *p*<0.001 | -4.632  *p*<0.001 | -1.575  *p=*0. 692 |
| Interpersonal | 116.664  *p*<0.001 | 1.508  *p*=0.790 | 8.361  *p*<0.001 | -8.553  *p*<0.001 | -6.326  *p*<0.001 | -6.654  *p*<0.001 | -0.730  *p=*1. 000 |
| Altruism | 114.459  *p*<0.001 | 0.496  *p=*1. 000 | 5.536  *p*<0.001 | -9.504  *p*<0.001 | -4.690  *p*<0.001 | -8.504  *p*<0.001 | -4.317  *p*<0.001 |
| Engagement | 206.132  *p*<0.001 | 3.374  *p=*0. 004 | 9.970  *p*<0.001 | -12.971  *p*<0.001 | -5.971  *p*<0.001 | -9.069  *p*<0.001 | -3.636  *p=*0. 002 |
| Self-competence | 237.316  *p*<0.001 | 9.576  *p*<0.001 | 6.165  *p*<0.001 | -14.871  *p*<0.001 | -3.783  *p*=0.001 | -5.027  *p*<0.001 | -9.086  *p*<0.001 |
| Optimism | 329.619  *p*<0.001 | 11.838  *p*<0.001 | 2.469  *p=*0. 081 | -15.466  *p*<0.001 | -9.503  *p*<0.001 | -3.459  *p=*0. 003 | -13.131  *p*<0.001 |
| Respect | 139.394  *p*<0.001 | 1.422  *p=*0. 093 | 7.262  *p*<0.001 | -10.506  *p*<0.001 | -5.382  *p*<0.001 | -8.579  *p*<0.001 | -3.706  *p*=0.001 |

Note: The overall test was conducted using the Kruskal-Wallis H test, with values representing *χ*² statistics. Values for post-hoc pairwise comparisons represent standardized effect sizes, with corresponding *p*-values presented below. *p*-values for post-hoc comparisons were adjusted using the Bonferroni correction. *p* < 0.05 indicates a statistically significant difference.

**Supplementary Table 4** Classification quality indices for the 4- and 5-class solutions

| **Class** | average posterior probabilities | **Classification Error Rate (%)** |
| --- | --- | --- |
| 4 | 0.862-0.926 | 7.4-13.8 |
| 5 | 0.878-0.929 | 7.1-12.2 |

**Supplementary Fig. 1 Five Latent profiles**
